# Cervical microbiota dysbiosis associated with high-risk Human Papillomavirus infection

**DOI:** 10.1371/journal.pone.0302270

**Published:** 2024-04-26

**Authors:** Natalia Zeber-Lubecka, Maria Kulecka, Michalina Dabrowska, Katarzyna Baginska-Drabiuk, Maria Glowienka-Stodolak, Andrzej Nowakowski, Aneta Slabuszewska-Jozwiak, Bożena Bednorz, Ilona Jędrzejewska, Magdalena Piasecka, Jolanta Pawelec, Elzbieta Wojciechowska-Lampka, Jerzy Ostrowski

**Affiliations:** 1 Department of Gastroenterology, Hepatology and Clinical Oncology, Centre of Postgraduate Medical Education, Warsaw, Poland; 2 Department of Genetics, Maria Sklodowska-Curie National Research Institute of Oncology, Warsaw, Poland; 3 Department of Cancer Prevention, Maria Sklodowska-Curie National Research Institute of Oncology, Warsaw, Poland; 4 First Department of Obstetrics and Gynecology, Centre of Postgraduate Medical Education, Warsaw, Poland; 5 Department of Lymphoid Malignancies, Maria Sklodowska-Curie National Research Institute of Oncology, Warsaw, Poland; University Federico II of Naples, ITALY

## Abstract

High-risk Human Papillomavirus (HR-HPV) genotypes, specifically HPV16 and HPV18, pose a significant risk for the development of cervical intraepithelial neoplasia and cervical cancer. In the multifaceted cervical microenvironment, consisting of immune cells and diverse microbiota, *Lactobacillus* emerges as a pivotal factor, wielding significant influence in both stabilizing and disrupting the microbiome of the reproductive tract. To analyze the distinction between the cervical microbiota and *Lactobacillus*-dominant/non-dominant status of HR-HPV and non-infected healthy women, sixty-nine cervical swab samples were analyzed, included 44 with HR-HPV infection and healthy controls. All samples were recruited from Human Papillomavirus-based cervical cancer screening program and subjected to 16s rRNA sequencing analysis. Alpha and beta diversity analyses reveal no significant differences in the cervical microbiota of HR-HPV-infected women, including 16 and 18 HPV genotypes, and those with squamous intraepithelial lesion (SIL), compared to a control group. In this study we identified significantly lower abundance of *Lactobacillus mucosae* in women with HR-HPV infection compared to the control group. Furthermore, changes in bacterial diversity were noted in *Lactobacillus* non-dominant (LND) samples compared to *Lactobacillus*-dominant (LD) in both HR-HPV-infected and control groups. LND samples in HR-HPV-infected women exhibited a cervical dysbiotic state, characterized by *Lactobacillus* deficiency. In turn, the LD HR-HPV group showed an overrepresentation of *Lactobacillus helveticus*. In summary, our study highlighted the distinctive roles of *L*. *mucosae* and *L*. *helveticus* in HR-HPV infections, signaling a need for further research to demonstrate potential clinical implications of cervical microbiota dysbiosis.

## Introduction

Persistent infections with high-risk Human Papillomavirus (HR-HPV) genotypes–HPV16 or HPV18 –constitute a major risk factor for cervical intraepithelial neoplasia (CIN) and cervical cancer (CC) [1]. HR-HPV infections were found in 99.7% of CC case studies [1]. The estimated lifetime risk of infection with any type of HPV is about 80%, while the risk of developing CC is only 0.6% [2]. In fact, about 90% of HR-HPV infections are transient and resolve spontaneously [3]. In recent years, preventing HPV infection has emerged as a major health priority [4]. Significant strides have been made in primary prevention, largely due to the introduction of HPV vaccines, which have demonstrated high efficacy in preventing infection. Studies suggest that transitioning from first-generation HPV vaccines to nonavalent vaccines could potentially enhance prevention rates for both low- and high-grade squamous intraepithelial lesions (L- and HSILs) in nearly 90% of cases [5].

The microbiota found across diverse areas of the female genital tract may display both similarities and notable distinctions [6–8]. The vaginal, cervical and cervico-vaginal microbiota collectively refer to the diverse array of microorganisms, encompassing bacteria, viruses, and fungi, residing in the vagina and on the cervix [9]. Several factors: hormonal fluctuations, sexual activity, hygiene practices, as well as contraceptive and antibiotic usage influence the composition of genital tract microbiota [10–12]. In contrast to the intestinal microflora, which is characterized by a large diversity of microorganisms, the genital tract of healthy women mainly contain one or several species of lactic acid bacilli, such as *Lactobacillus crispatus*, *L*. *gasseri*, *L*. *iners*, *L*. *jensenii* and L. acidophilus [13]. In turn, bacterial vaginosis is associated with the elimination of *Lactobacillus spp*. that become replaced by a diverse mix of anaerobic bacteria, such as *Anaerococcus*, *Atopobium*, *Dialister*, *Fusobacterium*, *Gardnerella*, *Gemella*, *Prevotella*, *Megasphaera*, *Parvimonas*, *Peptoniphilus*, and *Peptostreptococcus* [14]. *Lactobacillus spp*. play a range of important roles in the homeostasis of the cervical and vaginal microenvironment, such as anti-inflammatory effects that improve barrier function. *Lactobacillus* species produce lactic acid, which acidifies the local microenvironment to pH <4.5. protecting against the development of inflammation [15]. Moreover, metabolites produced by *Lactobacillus spp*. stimulate the production of antimicrobial peptides and anti-inflammatory cytokines [13].

The microbial communities found in the vagina and cervico-vaginal environment have been categorized into five major Community State Types (CSTs). However, the current CSTs classification offers only a partial comprehension of how the microbiota relates to cervico-vaginal conditions in women, largely due to limitations in bacterial identification technologies [16]. These categories are characterized by the predominance of specific *Lactobacillus* species. CST-I is primarily dominated by *L*. *crispatus*, CST-II by *L*. *gasseri*, CST-III by *L*. *iners*, and CST-V by *L*. *jensenii*. In contrast, CST-IV exhibits a more diverse microbial composition [17]. France et al. introduced the VALENCIA (Vaginal Community State Type Nearest Centroid) classifier tool, which enables consistent assignment of CSTs within the vaginal microbiota of reproductive-age women [18].

In women diagnosed with HR-HPV infection, long-term alterations in the cervico-vaginal microbiota composition positively correlated with microbial diversity at two time points, six months apart [16]. Women who initially exhibited a high abundance of *L*. *iners* tended to maintain a more stable microbiome composition in subsequent visits compared to those with communities depleted of *Lactobacillus* at the outset. Certain species including *L*. *acidophilus* and *Megasphaera genomosp type 1* were linked to changes in CSTs between visits [16]. Persistent HPV infection and CIN development were associated with higher abundance of *Gardnerella*, *Prevotella*, and *Megasphaera* in cervical and cervico-vaginal microbiota [19–21]. Moreover, some research revealed a trend of co-infection between HPV and syphilis, caused by *Treponema pallidum*, in certain populations [22]. In vitro experiments have shed light on potential mechanisms by which microbiota influence HPV infections, including the disruption of the cervical epithelial barrier through the regulation of adherence junction proteins, modulation of cervical immune responses, and alteration of miRNA expression [19]. However, SILs and progression to precancerous interepithelial neoplasia and CC are the clinical outcomes of HPV infection, the role of cervical dysbiosis in tumorigenesis remains unclear [14, 23–29].

Considering all the mentioned dependencies, in this study we conducted a comparison between a group of women infected with HR-HPV and non-infected healthy women to identify differences in cervical microbiota profiles according to the HPV infection and *Lactobacillus* non-dominant status using 16S rRNA gene amplicon sequencing.

## Materials and methods

### Patients and controls recruitment

The study was conducted according to the guidelines of the 1964 Declaration of Helsinki and approved by the Bioethics Committee at the Maria Sklodowska-Curie National Research Institute of Oncology in Warsaw Review Board (No 42/2021). All participants provided informed consent to participate. Between 2^nd^ August 2021 and 30^th^ September 2022 enrolled HPV-infected and healthy women were recruited under the Human Papillomavirus-based cervical cancer screening program in the Department of Cancer Prevention, Maria Sklodowska-Curie National Research Institute of Oncology. The study cohort included Polish Caucasian women who had provided written informed consent before being included in the study. Inclusion criteria for the study encompassed women older than 18 years, who were sexually active and unvaccinated for HPV. None of the participants were undergoing hormone replacement therapy. Exclusion criteria consisted of pregnancy and lactation, a history of resection surgery, hysterectomy, or pelvic radiotherapy, HIV infection, vaginal douching, or antibiotic therapy.

### Sample collection procedure

#### Papanicolaou test (Pap test) examination and HPV DNA testing

During the cervical screening visit, the patients underwent Pap test examination first. Brushes with exfoliated cervical cells preserved in 20 ml SurePath® Preservative Solution were subjected to liquid-based cytology and testing for the presence of HPV DNA. HPV infection was assayed using the Cobas4800HPV test for 3 HR signals of HPV types 16, 18 and others (Roche Molecular Diagnostics, Pleasanton, CA). A microscopic screening for precancerous cervical lesions based on the Bethesda System which divides epithelial cell abnormalities into low- and high-grade squamous intraepithelial lesion (L/HSIL) categories. CIN 1 and CIN 2/3 were classified as LSIL and HSIL, respectively. Additionally, equivocal morphological features including atypical squamous cells (ASCs) were subdivided into two categories: ASC of Undetermined Significance (ASC-US) and ASC of HSIL (ASC-H).

#### Cervical swab samples collection, DNA extraction and 16S rRNA sequencing for microbiota analysis

After Pap test examination, cervical swab samples were collected aseptically from each patient using 4N6FLOQSwabs™ (Thermo Fisher Scientific, MA, USA), transferred to a collection tube and stored at −80°C within 30 minutes of collection. Bacterial genomic DNA was extracted from 4N6FLOQSwabs™ (Thermo Fisher Scientific, MA, USA) samples using the PureLink™ Microbiome DNA Purification Kit, according to the manufacturer’s instructions. The quantity and purity of extracted DNA were measured with the Nanodrop ND-1000 spectrophotometer (Thermo Fisher Scientific, MA, USA). Cervical microbiota analysis was performed using the Ion Torrent technology, following the taxonomic identification of bacteria based on hypervariable fragments of the 16S rRNA gene sequencing (Ion 16S™ Metagenomics Kit; Thermo Fisher Scientific, MA, USA) as described previously [30].

#### Statistical and bioinformatic analysis

Unmapped BAM files were converted to FASTQ using Picard’s SamToFastq. Additional steps of the analysis were performed using Mothur software version 1.43 [31]. FASTQ files were converted to the FASTA format. We only included sequences that were 200–300 bases in length, with an average base quality of 20 in a sliding window of 50 bases, and a maximum homopolymer length of 10. Chimeric sequences were identified with the vsearch algorithm using the default parameters, with internal sequence collection as the reference database [32]. Chimeric sequences were removed and the remaining 16S rRNA sequences were classified using the Wang method and the SILVA [33] bacterial 16S rRNA database for reference (release 138). The bootstrap cut-off was 80%. Alpha diversity analysis was performed using the Shannon and Chao index as an indicator. Principal coordinate analysis (PCoA) was performed using the Bray-Curtis index as a distance measure. The ANOSIM was used to test the significance of clustering patterns. The differential abundance of taxa was assessed using the LinDA with the default parameters [34]. The Mann-Whitney U-test was utilized to assess variations in diversity indices between control and HR-HPV samples, while the Wilcoxon paired test was employed to identify statistically significant differences within paired patient samples. Adjusted p-values below 0.05, after controlling for false discovery rate (FDR), were deemed as indicative of significance. *Lactobacillus* species was determined by reassigning reads to the Greengenes database, version 13_8 [35]. The patients were defined as *Lactobacillus*-dominant if the bacteria abundance was higher than 70% [36].

## Results

### Patients and samples, HPV genotype testing and histopathologic diagnoses

Ultimately, 46 women diagnosed with a HR-HPV infection (median age 40 years, range 25–59) were enrolled in the study. The control group comprised 27 healthy women (median age 42 years, range 27–58) who tested negative for HPV and exhibited normal Pap tests. Nevertheless, the quantity and quality of DNA isolated from cervical swab samples enabled the construction of libraries from 44 HR-HPV-positive and 25 HPV-negative samples. Therefore, in the final analysis, a total of 69 samples were utilized for the study of cervical microbiota.

The characteristics of HR-HPV-infected women according to the Bethesda System is presented in Table 1. In this group, 13 infected women were diagnosed with SIL (and 3 of them were HSIL). Three HR-HPV infected women with SIL also presented ASC-US features. ASC-US and ASC-H were detected in 4 and 1 women without SIL, respectively. No-intraepithelial lesions (NILM) without SIL or ASC findings were shown in another 26 HR-HPV- infected women. The Pap test was normal in all HPV-negative women. Two HPV-infected women used intrauterine contraceptive devices (IUD).

**Table 1:** Characteristics of total HR-HPV-infected women based on Pap tests results according to the Bethesda system.

HR-HPV infected women (n = 44)	
16 types	10 (22.7%)
18 types	4 (9.1%)
HR-HPV variants other than 16 and 18	30 (68.2%)
HR-HPV infected women with SIL: n = 13 (29.5%) from total 44	
LSIL	10 (22.7%)
HSIL	3 (6.8%)
HR-HPV infected women without SIL who additionally presented equivocal morphological features: n = 5 (11.4%) from total 44	
ASC-US	4 (9.1%)
ASC-H	1 (2.3%)
NILM without SIL, ASC-US and ASC-H: n = 26 (59.1%) from total 44	

HR-HPV, high-risk Human Papillomavirus; SIL, squamous intraepithelial lesion; LSIL, low-grade squamous intraepithelial lesion; HSIL, high-grade squamous intraepithelial lesion; ASC, atypical squamous cells; ASC-US, Undetermined Significance (ASC-US); ASC-H, atypical squamous cells cannot exclude HSIL; NILM, No-intraepithelial lesions.

### Cervical microbiota diversity

**Fig 1:**
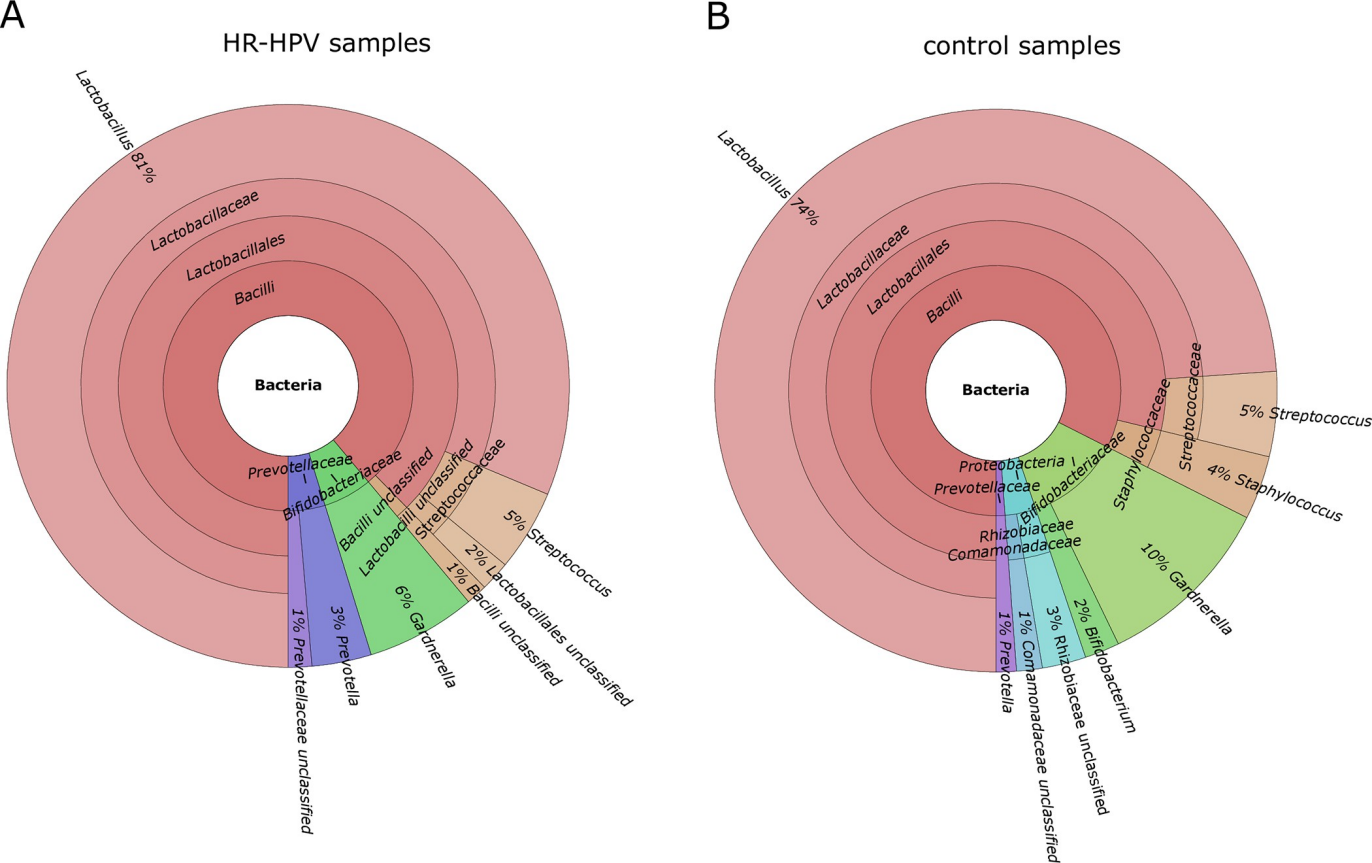
Krona charts of the taxon abundance with a mean >1% of the total reads found in the HR-HPV (A) and control (B) samples.

**Fig 2:**
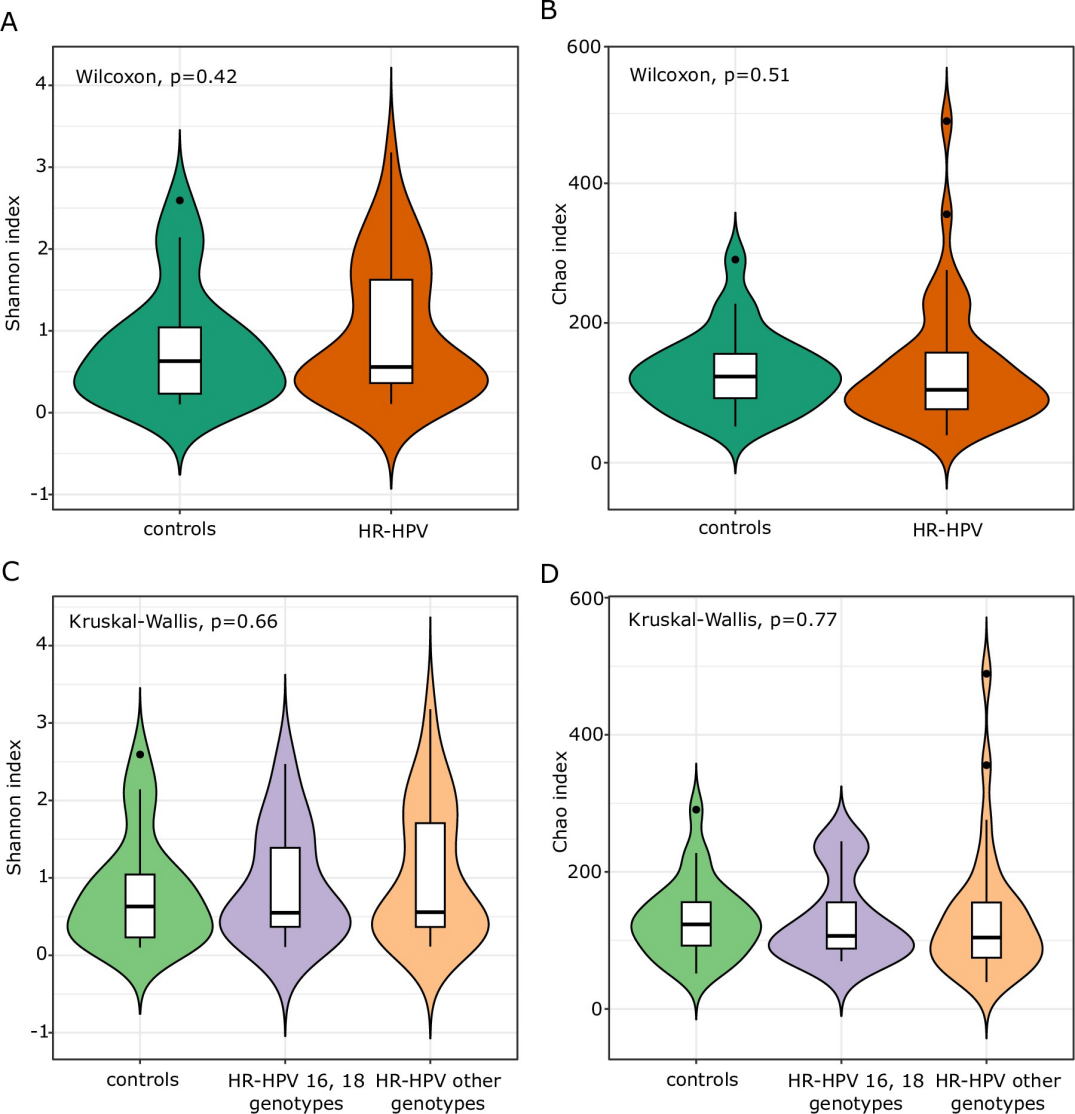
Shannon index (A, C), and Chao index (B, D) of the cervical microbiota between the HR-HPV, 16 and 18 or other HR-HPV genotypes and control group.

**Fig 3:**
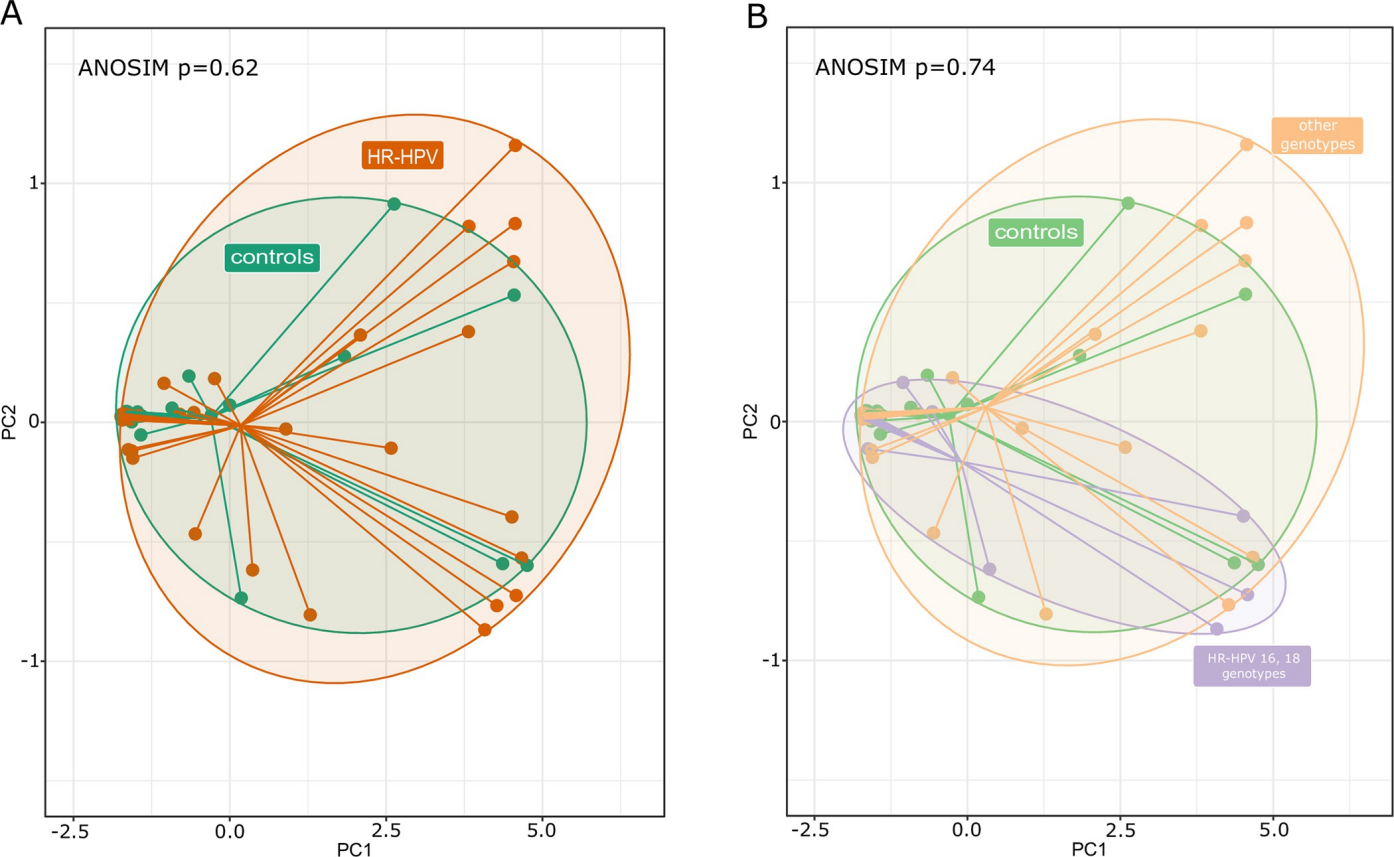
Principal coordinates analysis based on the Bray–Curtis index for HR-HPV (A), 16 and 18 or other HR-HPV genotypes (B) and control group.

Of 894 taxa (131 of which were found in over 0.01% of reads), 454 taxa were present in both groups, and 376 and 64 were detected only in HR-HPV patients and healthy controls, respectively (Fig 1). In total, we detected an average of 100 taxa per sample and 178517 reads. At the OTU level, microbial richness, and alpha and beta diversity were estimated using Chao, Shannon and PCoA indices, respectively. Alpha diversity analysis showed no statistically significant differences in the cervical microbiota of women infected with HPV compared to the control group (Fig 2A and 2B). A similar result was obtained after clustering the HPV types, HR-HPV 16 and 18, compared to the group of patients infected with other HR-HPV (Fig 2C and 2D). Also, PCoA showed no statistical differences in the beta diversity of cervical microbiota between HR-HPV and controls (Fig 3A). A comparable result was obtained comparing 16 and 18 HR-HPV patients to other HR-HPV and controls (Fig 3B).

At the genus level, only unclassified *Comamonadaceae* differentiated HR-HPV-infected patients from controls (padj <0.05) whose lower amounts were found in HR-HPV samples. The abundance of other species, unclassified *Rhizobiaceae* and *Aminobacter*, tended to be statistically significant and was also lower in HR-HPV women (Table 2).

**Table 2:** Bacteria at the genus level differentiating HR-HPV and control samples.

Taxa	baseMean	log2 FC	lfcSE	stat	p_value_	p_adj_
*Comamonadaceae unclassified*	579.059	-3.900	0.836	-4.667	1.513 × 10^−5^	4 × 10^−3^
*Rhizobiaceae unclassified*	675.068	-3.552	0.973	-3.649	5.1482 × 10^−4^	6.5 × 10^−2^
*Aminobacter*	38.768	-1.840	0.540	-3.407	1.11461 × 10^3^	9.3 × 10^−2^

baseMean, the average of the normalized count values, divided by size factors, taken over all samples; log2FC log2 fold change between the groups; lfcSE, standard error of the log2FC estimate; stat, the value of the test statistic; pv_alue_, p-value of the test; p_adj_, Benjamini–Hochberg-adjusted p-value.

**Fig 4:**
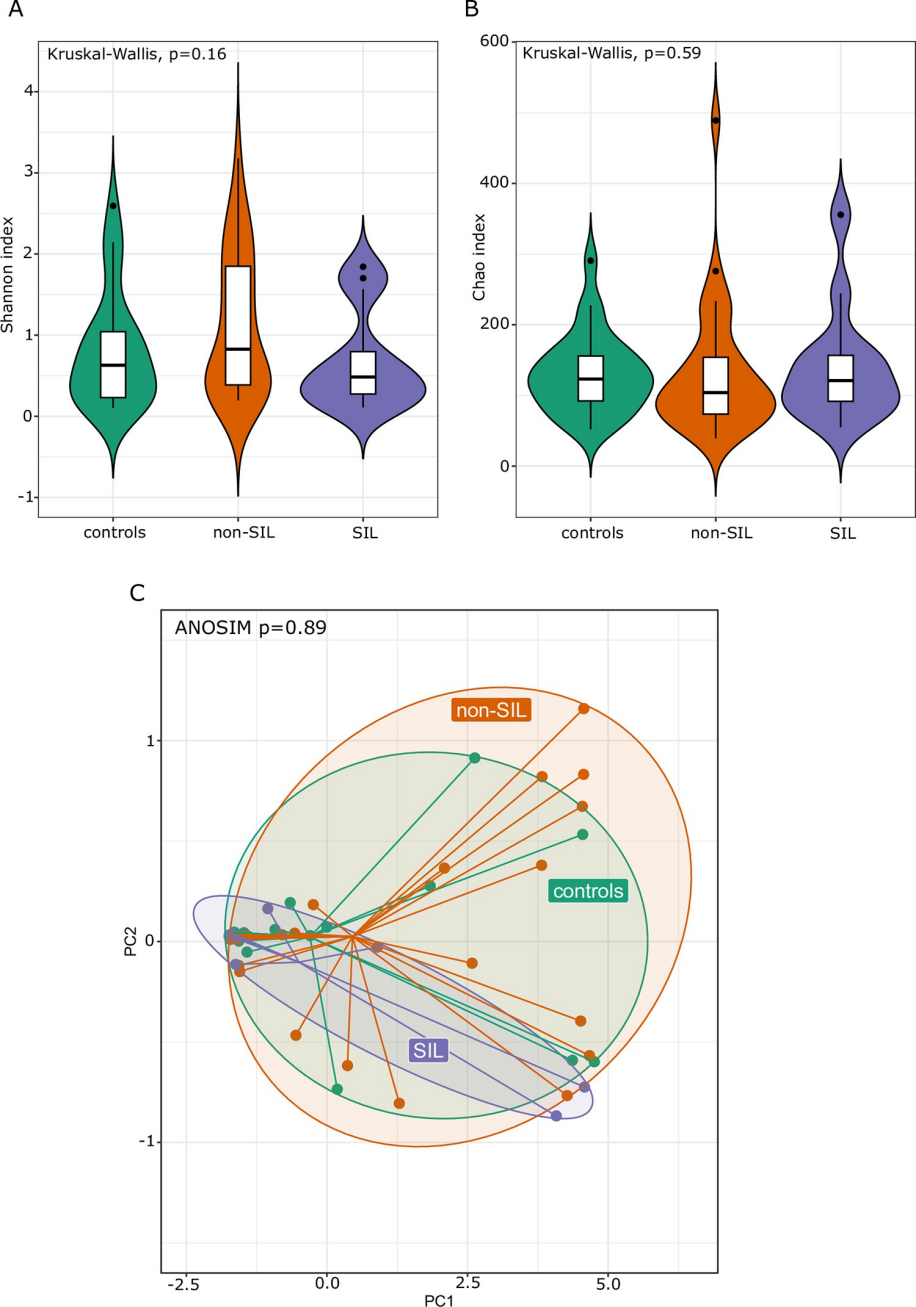
Shannon index (A), Chao index (B) and Principal coordinates analysis based on the Bray–Curtis index (C) of the cervical microbiota between the HR-HPV with and without squamous intraepithelial lesion (SIL) and control group.

Next, we compared the samples from HR-HPV-positive women with or without SIL and those from the healthy controls. Again, both alpha (Fig 4A and 4B) and beta diversity (Fig 4C) of cervical microbiota did not differ between the studied groups. Due to the limited size of the patient group with Pap-test lesions, statistically significant results were not attainable. The only difference related to the significantly lower abundance of unclassified *Comamonadaceae* genus was demonstrated in non-SIL patient samples compared to controls.

### Lactobacilli findings

**Fig 5:**
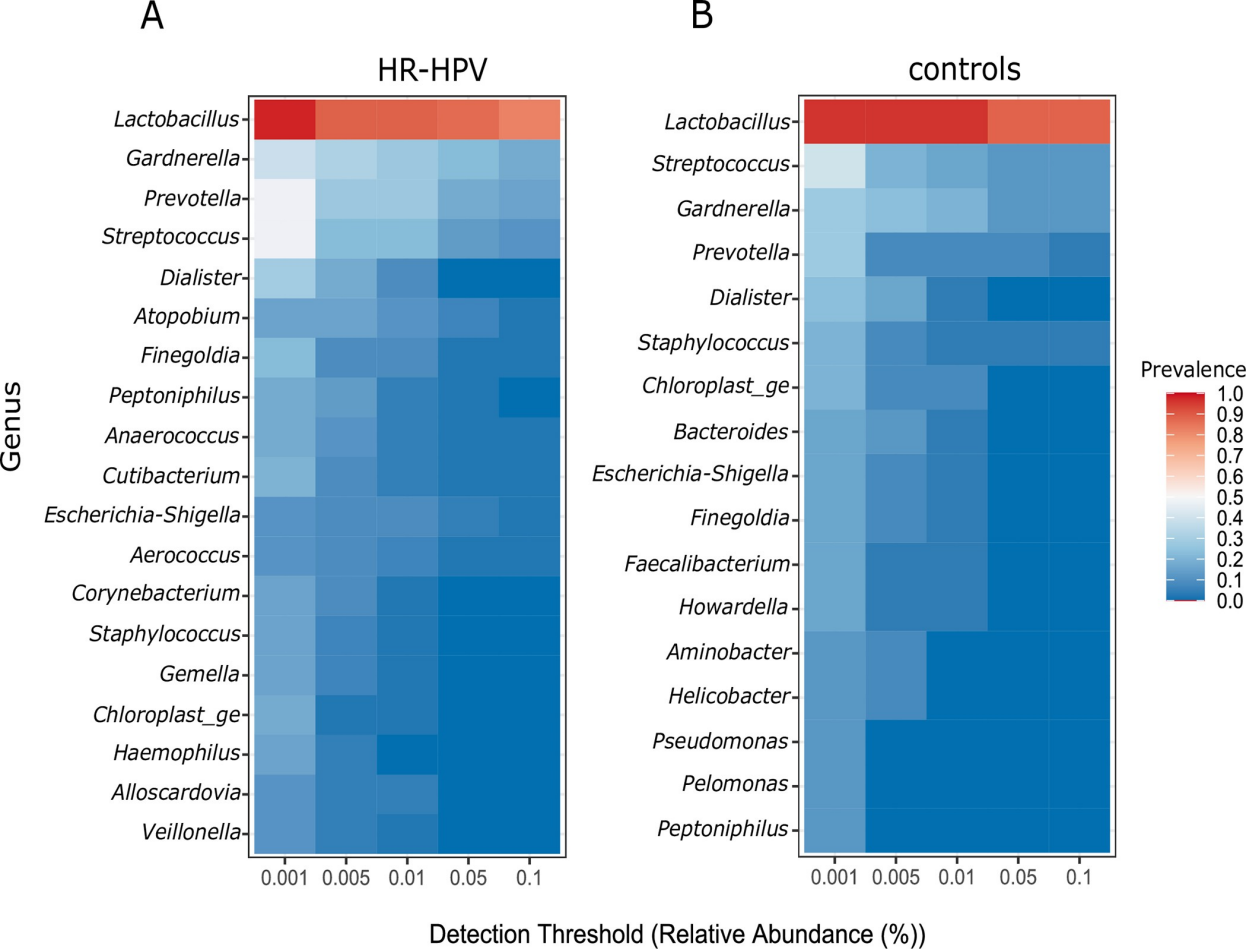
Heat-map diagram of the cervical microbiota composition at genus level for HR-HPV (A) and control (B) groups.

*Lactobacillus* species were found in all 25 HPV-negative and in 41 out of 44 HR-HPV-positive women (Fig 5A and 5B, respectively). Of these, *Lactobacillus* dominated (with the bacteria abundance higher than 70%) in the cervical microbiota of 17, from total 25 HPV-negative and 27, from total 44 HR-HPV-positive women. The distribution of predominant *Lactobacillus* species was presented in Table 3.

**Table 3:** *Lactobacillus* distribution in *Lactobacillus*-dominant HR-HPV-positive and HPV-negative samples (lactobacilli threshold >70%).

*Lactobacillus* classification	Samples number (%)	*Lactobacillus* % range	*Lactobacillus* % median	*Lactobacillus* % sd	p_val_
HR-HPV samples (N: 44 (100%)) with:					
*Lactobacillus*-dominant (total lactobacilli reads)	**27 from total 44 HR-HPV** (61.36)	74.11–99.89	97.56	6.69	0.791
*Lactobacillus unclassified*	26/27 (96.30)	40.85–99.98	54.99	28.86	0.920
*Lactobacillus helveticus*	23/27 (85.19)	1.97–54.31	34.14	17.32	0.920
*Lactobacillus iners*	17/27 (62.96)	0.16–100	38.18	42.48	0.435
HR-HPV SIL samples (N: 13/44 (29.55%)) with:					
*Lactobacillus*-dominant (total lactobacilli reads)	**10 from total 13 HR-HPV SIL** (76.92)	83.20–99.81	98.10	4.99	-
HPV- negative (control) samples (N: 25 (100%)) with:					
*Lactobacillus*-dominant (total lactobacilli reads)	**17 from total 25 HPV- negative** (68.0)	75.69–99.66	97.90	6.75	0.791
*Lactobacillus unclassified*	17/17 (100.0)	0.16–100	22.77	29.31	0.920
*Lactobacillus helveticus*	14/17 (82.35)	5.54–92.54	53.88	29.82	0.920
*Lactobacillus iners*	7/17 (41.18)	1.06–99.84	84.29	48.21	0.435

p_val_. the p-value for the chi-square test; bolded, the *Lactobacillus*-dominant samples based on total lactobacilli reads.

Seventeen HR-HPV-positive samples and 8 HPV-negative samples were classified as *Lactobacillus* non-dominant (LND). Several pathogenic bacteria were detected in HPV-positive and LND samples. They were classified as CST type IV, including *Streptococcus*, *Prevotella*, *Gardnerella*, *Streptococcus*, *Atopobium*, *Megasphaera* and *Anaerococcus* genera (Table 4). In HPV-negative and LND samples, we identified *Gardnerella*, *Prevotella* and *Streptococcus* in 4, 2 and 1 samples, respectively.

**Table 4:** Bacterial distribution in *Lactobacillus* non-dominant HR-HPV-positive and control samples.

Bacteria classification	Samples number (%)	Bacteria % range	Bacteria % median	Bacteria % sd	p_val_
non-*Lactobacillus* dominant HR-HPV samples (N: 17/44 (38.64%))					
Without *Lactobacillus*	3/17 (17.65)	-	-	-	-
*Lactobacillus*	14/17 (82.35)	0.38–69.35	21.07	21.59	0.078
*Streptococcus*	8/17 (47.05)	3.16–70.24	11.00	30.98	-
*Prevotella*	12/17 (70.59)	1.70–37.31	15.19	11.56	-
*Gardnerella*	8/17 (47.06)	1.03–51.53	26.50	17.67	-
*Atopobium*	3/17 (17.64)	0.82–27.49	7.53	13.87	-
*Megasphera*	3/17 (17.64)	4.07–10.04	6.94	2.99	-
*Anaerococccus*	2/17 (11.76)	1.86–13.16	7.51	7.99	-
non-*Lactobacillus* dominant control samples (N: 8/25 (32%))					
*Lactobacillus*	8/25 (32.0)	0.72–69.44	33.24	29.84	0.078
*Streptococcus*	1/8 (12.5)	64.88	-	-	-
*Prevotella*	2/8 (25.0)	9.24–10.44	9.84	0.85	-
*Gardnerella*	4/8 (50.0)	1.54–93.07	25.27	40.11	-
*Atopobium*	None				
*Megasphera*	None				
*Anaerococccus*	None				

p_val_. the p-value for the chi-square test

When the cervical microbiota of HR-HPV-infected women was compared to those of control group, one species of lactobacilli, *L*. *mucosae* was found to be less represented in the HR-HPV group (p_adj_ 0.02; FC -2.37). In contrast, no differentiating lactobacilli were found in the cervical microbiota of women with SIL compared to both the control and HR-HPV non-SIL groups.

### Cervical microbiota dominated vs non-dominated by *Lactobacillus*

**Fig 6:**
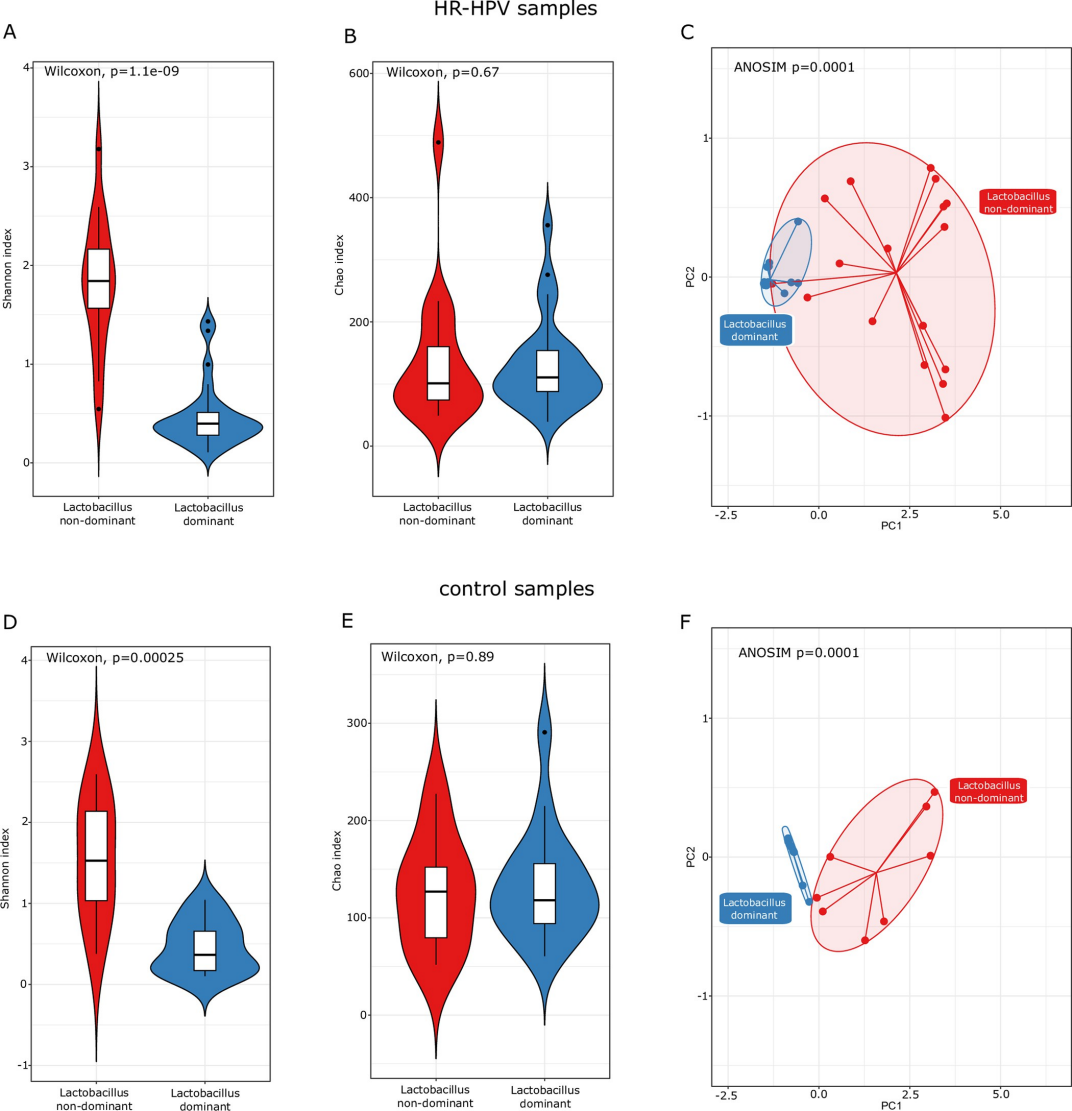
Shannon index (A, D), Chao index (B, E) and Principal coordinates analysis based on the Bray–Curtis index (C, F) in comparisons of *Lactobacillus*-dominant (LD) vs. *Lactobacillus* non-dominant (LND) of HR-HPV and control groups.

In both LND groups of women infected with HR-HPV and controls we observed a higher bacterial diversity compared to the LD (*Lactobacillus*-dominant) group (Shannon index p_adj_ 1.1e-09 and 0.00025, respectively) (Fig 6A and 6D). Furthermore, the groups also differed as regards the beta diversity level (ANOSIM p = 0.0001, R = 0.703; ANOSIM p = 0.0001, R = 0.789) (Fig 6C and 6F) but did not differ as regards bacterial richness (Fig 6B and 6E). The abundance of 23 taxa significantly differentiated LD from LND samples in women infected with HR-HPV (Table 5). Among these, 5 and 18 taxa were under- and overrepresented, respectively in LND HR-HPV samples.

**Table 5:** Taxa distinguishing LD from LND samples in women infected with HR-HPV.

Taxa	baseMean	log2 FC	lfcSE	stat	p_value_	p_adj_
*Lactobacillus*	902979.500	**2.343**	0.462	5.067	8.55 × 10^−6^	4.17 × 10^−4^
*Lactobacillaceae unclassified*	189.545	**1.492**	0.322	4.632	3.48 × 10^−5^	1.063 × 10^−3^
*Firmicutes unclassified*	15492.930	**1.026**	0.310	3.316	1.891 × 10^−3^	2.4279 × 10^−2^
*Bacilli unclassified*	21988.880	**0.972**	0.282	3.452	1.282 × 10^−3^	1.8405 × 10^−2^
*Lactobacillales unclassified*	33606.080	**0.877**	0.282	3.112	3.338 × 10^−3^	3.7017 × 10^−2^
*Aerococcaceae unclassified*	16.648	-0.863	0.256	-3.376	1.592 × 10^−3^	2.1575 × 10^−2^
*Salmonella*	8.499	-1.039	0.341	-3.049	3.963 × 10^−3^	4.2042 × 10^−2^
*Peptostreptococcales-Tissierellales unclassified*	26.816	-1.169	0.323	-3.619	7.88 × 10^−4^	1.2017 × 10^−2^
*Actinobacteria unclassified*	222.009	-1.303	0.319	-4.087	1.93 × 10^−4^	4.28 × 10^−3^
*Veillonellales-Selenomonadales unclassified*	23.992	-1.318	0.284	-4.637	3.43 × 10^−5^	1.063 × 10^−3^
*Coriobacteriales unclassified*	21.529	-1.413	0.453	-3.118	3.282 × 10^−3^	3.7017 × 10^−2^
*Actinobacteriota unclassified*	49.606	-1.461	0.364	-4.017	2.39 × 10^−4^	4.851 × 10^−3^
*Clostridia unclassified*	82.038	-1.489	0.388	-3.835	4.15 × 10^−4^	7.24 × 10^−3^
*Bacteroidia unclassified*	5.314	-1.599	0.378	-4.237	1.21 × 10^−4^	3.287 × 10^−3^
*Bacteroidales unclassified*	38.783	-1.696	0.351	-4.826	1.87 × 10^−5^	7.6 × 10^−4^
*Peptostreptococcales-Tissierellales_fa unclassified*	42.686	-1.727	0.422	-4.098	1.86 × 10^−4^	4.28 × 10^−3^
*Peptoniphilus*	130.802	-1.757	0.552	-3.183	2.746 × 10^−3^	3.35 × 10^−2^
*Bifidobacteriaceae unclassified*	74.294	-1.841	0.485	-3.794	4.69 × 10^−4^	7.625 × 10^−3^
*Streptococcaceae unclassified*	193.501	-2.166	0.544	-3.980	2.67 × 10^−4^	5.014 × 10^−3^
*Veillonellaceae unclassified*	57.519	-2.492	0.445	-5.596	1.51 × 10^−6^	1.23 × 10^−4^
*Prevotellaceae unclassified*	1035.783	-3.003	0.509	-5.898	5.57 × 10^−7^	8 × 10^−5^
*Prevotella*	2762.131	-3.177	0.543	-5.849	6.56 × 10^−7^	8 × 10^−5^
*Dialister*	161.618	-3.217	0.622	-5.173	6.06 × 10^−6^	3.7 × 10^−4^

baseMean, the average of the normalized count values, divided by size factors, taken over all samples; log2FC log2 fold change between the groups; lfcSE, standard error of the log2FC estimate; stat, the value of the test statistic; pv_alue_, p-value of the test; p_adj_, Benjamini–Hochberg-adjusted p-value; bolded, taxa underrepresented in *Lactobacillus* non-dominant (LND) HR-HPV samples.

Notably, a similar analysis conducted in the control group revealed only one statistically significant taxon (*Bifidobacteriaceae* unclassified, p_adj_ 0.046) distinguishing LD from LND. Even though *Lactobacillus helveticus* was overrepresented in LD HR-HPV group (FC 2.53; p_adj_ 0.029) as compared to LND HR-HPV group, no distinguishing lactobacilli were found by the same comparisons in the control group.

## Discussion

The cervicovaginal mucosa relies on several key defense mechanisms, including antimicrobial peptides and microbiota primarily composed of lactobacilli. Any disruptions to these protective mechanisms may lead to physical and chemical changes that harm the cervical epithelium and vaginal mucosa [37]. Specifically, a reduction in lactobacilli, which are responsible for producing lactic acid, can promote abnormal bacterial growth and reduce the presence of protective flora, thereby weakening the body’s natural defense system against viral infections [38].

Several studies have explored the potential influence of HPV infection on the cervico-vaginal microbiota and vice versa [39–41]. However, exact mechanisms and causal relationships are still being elucidated. Dysbiosis, or imbalance, in the cervico-vaginal microbiota has been associated with HPV persistence, which is a key factor in the development of cervical precancerous lesions and cancer [42]. Certain bacteria stimulate immune cells and modulate inflammation, potentially affecting the clearance or persistence of HPV [43]. While HPV vaccination has reduced the burden of CC, non-vaccine-preventable HPV genotypes still pose a risk [44, 45]. Crucially, not every woman infected with HR-HPV develop CC, leading to investigations into potential protective factors. The hypotheses imply that beneficial bacteria, including *L*. *acidophilus* in the cervico-vaginal microbiota, could contribute to safeguarding against CC development among HR-HPV-infected women [46]. Detailed microbiome profiling in a Dutch cervical cancer screening program found that women with typical cervical smears and a higher abundance of *L*. *acidophilus* were associated with a lower risk of HSIL, highlighting the role of this bacteria in cervico-vaginal microbiota dynamics and continuity [46].

The primary objective of this study was to investigate the variances in cervical microbiota profiles between two groups: women infected with HR-HPV and non-infected healthy women. We aimed to discern distinctions in cervical microbiota characteristics concerning HR-HPV infection and the prevalence of non-dominant *Lactobacillus* species.

The analysis of alpha and beta diversity revealed no significant differences in the cervical microbiota of women infected with HPV, including HR-HPV types 16 and 18, as well as the SIL, compared to the control group. A limitation of this study is related to the fact that only 10 LSIL and 3 HSIL samples were included. Unclassified *Comamonadaceae* were the differentiating bacteria of the HR-HPV groups and controls, SIL and non-SIL. It seems that the occurrence of these differentiating bacteria in the cervix was related to HPV infection but not to SIL. A lower abundance of *Comamonadaceae* in HR-HPV-infected women was also observed in two studies conducted by Chen et al. [47, 48].

Liu et al. showed that the Shannon index of vaginal microbiota diversity was higher in women with HPV infection, signifying a greater number of bacteria compared to HPV-negative women [49]. Moreover, a lower Simpson’s index in HPV-positive women indicated the complexity of the vaginal microflora enhanced by HPV infection but reduced under stable healthy vaginal conditions. However, the bacterial richness did not show a significant relationship with HPV infection. As shown in previous studies, significant differences in cervical microbiota diversity were only observed in HSIL [41, 50]. Guo et al. reported a significantly higher alpha diversity in the HPV+HSIL group compared to the HPV+LSIL group [41]. Nevertheless, no significant differences were found between HPV+LSIL and HPV+NoSIL, nor between HPV+NoSIL and HPV-negative groups. Additionally, PCoA revealed only slight variations in microbial samples among the HPV-negative, HPV+NoSIL, HPV+LSIL, and HPV+HSIL groups [41]. Lin et al. demonstrated no significant changes in Shannon diversity for CIN in the vagina and cervix, even though the number of observed OTUs in the cervical specimens was slightly higher for CIN than in healthy controls [50]. However, microbial diversity increased significantly in cervical specimens compared to their corresponding vaginal specimens in CIN. Furthermore, when examining different subtypes of CIN, the Shannon diversity in the cervical microbiota significantly increased in the CIN 1 subtype. Additionally, *Comamonas (Comamonadaceae)*, *Rhizobium (Rhizobiaceae)*, and *Pseudomonas (Pseudomonadaceae)* were identified as bacteria strongly linked with CIN in the cervix [50].

The most remarkable changes in bacterial diversity affect women who have developed CC. In a case-control analysis involving healthy individuals and patients diagnosed with either CIN 2/3 or invasive CC, microbial richness was notably elevated in that group compared to the controls [51]. The increase was associated with a higher count of OTUs. Our previous study [30] revealed that cervical microbiota diversity measured by the Shannon index was significantly greater in pre-treatment CC women in comparison with control samples. However, we did not observe significant differences in species richness. These results reveal significant alterations in the cervical microbiota of CC patients relative to that in healthy controls.

It is the first study which revealed that among various lactobacilli studied, only *L*. *mucosae* exhibited a significantly lower presence in the group of women with HR-HPV infection compared to the control group. In contrast, our analysis did not reveal any significant differences in the composition of lactobacilli in the cervical microbiota of women with SIL when compared to both the control group and the HR-HPV non-SIL group. *L*. *mucosae* inhabits the digestive systems of both humans and animals, and it is also found in the human vaginal environment. *L*. *mucosae* produces exopolysaccharides (EPS), which exhibit anti-inflammatory effects [52]. This bacterium is known for its ability to neutralize toxins and exhibit antimicrobial activity, effectively controlling harmful substances including Zen toxin and inhibiting various Gram-positive and Gram-negative pathogens, including *Escherichia coli*, *Salmonella typhimurium* and *Staphylococcus* [53, 54]. As of now, the presence of *Lactobacillus mucosae* in the cervix has not been described or documented in scientific literature. Moreover, previous research indicated that *Lactobacillus mucosae* is not commonly found in the vaginal microbiota [55]. Das Purkayastha et al. found a relative dominance of vaginal *L*. *mucosae* compared to *L*. *crispatus* and *L*. *jensenii* among both non-pregnant and pregnant women of Northeast India [56]. The authors suggested that *L*. *mucosae* might have a competitive advantage over other bacteria in terms of binding to receptors and utilizing nutrients within the vaginal epithelial cells [56]. *L*. *mucosae* was documented to produce the Lam29 protein which allowed it to effectively outcompete pathogens, safeguarding the intestinal mucosa [57]. Similarly, in the human vaginal environment, the epithelial layer serves as a protective barrier that pathogens seek to breach, while *Lactobacillus* aims to protect it by adhering and outcompeting the intruders. Since a lower abundance of *L*. *mucosae* may be a result of HPV infection, we hypothesize that a loss or decrease in the abundance of *L*. *mucosae* may lead to deficiencies in its protective function among HPV-infected women.

In both HR-HPV-infected and control groups, changes in bacterial diversity were observed in LND samples compared to LD. Such a result is consistent with previous studies [58–60]. Additionally, LND samples in HR-HPV-infected women were classified as CST IV, characterized as a dysbiotic state which includes *Lactobacillus* deficiency and consists of a diverse group of microorganisms, including both strictly obligate and facultative anaerobes [17, 61, 62]. Conversely, the LD HR-HPV group showed an overrepresentation of *L*. *helveticus*, which produces hydrogen peroxide, impedes the attachment of pathogens, and decreases the viability of bacteria associated with bacterial vaginosis [63]. During both the follicular and luteal phases, *L*. *helveticus* emerged as the predominant species [64]. Kim et al. confirmed the potential of *L*. *helveticus* HY7801 as a beneficial probiotic for the physiological vaginal state [65]. *In vivo* experiments showed that HY7801 reduced the presence of *Gardnerella vaginalis* and pro-inflammatory cytokines in BV-induced mice, improving histological changes of vaginal tissues. Importantly, it was found that HY7801 could alleviate bacterial vaginosis by inhibiting the virulence factor genes of *Gardnerella vaginalis* related to cell adhesion and biofilm formation, along with antibacterial activity against *G*. *vaginalis* [65]. Moreover, Scibior-Bentkowska et al. showed that an increased abundance of *L*. *helveticus*, *L*. *suntoryeus*, and *L*. *vaginalis* in women with persistent HR-HPV infection was associated with a potentially protective effect against the development of CIN 3 [66].

## Conclusions

Our study provided insights into the association between the cervical microbiota, HR-HPV infections, and *Lactobacillus* non-dominant status. We showed for the first time that only *L*. *mucosae* exhibited a significantly lower abundance in the group of women with HR-HPV infection in comparison with the controls. Alternatively, further research is needed before potential clinical implications of cervical microbiota dysbiosis could be generalized.
